# Philadelphia Letter

**Published:** 1869-07-01

**Authors:** Geo. R. Welding

**Affiliations:** 1372 N. Eleventh St., Philadelphia


### ORIGINAL COMMUNICATIONS.

EDITOR CHICAGO MEDICAL JOURNAL:

DEAR SIR—In the May number of your valuable paper a letter appears signed "Geo. J. Zeigler, M.D.," in reference to nitrous oxide.

As my alleged connection with this person in regard to the preparation of this article has caused me considerable chagrin, you must allow a professional brother a little space in your paper, to relieve himself from the odium which has been fastened upon him.

About eighteen years ago my acquaintance began with Mr. Zeigler, who was then employed in the hair-dressing establishment of Mr. Pollard, in this city, and I lost sight of him for some years, when my acquaintance was renewed with him, he being, at that time, pulling teeth and shaving, at Tenth and Arch streets.

Mr. S. S. White solicited me to assist Zeigler in procuring a *patent* upon nitrous oxide, and, notwithstanding my earnest protestations, did actually proceed to Washington, apply for a patent, procure machinery, etc., at an expense of over $1,000, and in order to achieve success (at the command of Zeigler) began experimenting upon dogs, having, forty dogs at one time in the back room of his building at Arch and Sixth streets. The place was called a "Dog Hospital." It was a cruel way to treat the unfortunate canines. They were all suspended by their tails, and he (Zeigler) injected the gas *per anum.*

To those who know me intimately, I need not say that I would not do any thing so unprofessional as to attempt to secure a *patent* upon ideas as old as Sir Humphrey Davy.

*I deny, most emphatically*; I owe it to my professional character to do so, that I gave any counsel to either Zeigler or White in this business! On two or three occasions White induced me to re-write and re-spell Zeigler's articles. I did not receive one cent pay for it. Mr. Zeigler is, as far as I know, a worthy mechanic. "Curling Hair" is a respectable occupation when legitimately performed. But I do most heartily protest against the charge of assisting either Ziegler or White in their operations.

Yours, truly,

GEO. R. WELDING, M.D.

1372 N. Eleventh St., Philadelphia.

